# Draft Genome Sequence of Lactococcus lactis Strain PrHT3, Isolated from Organic Basil

**DOI:** 10.1128/mra.00629-22

**Published:** 2022-11-07

**Authors:** Chelsea Truitt, Guadalupe Meza, Hung King Tiong, Peter R. Hoyt, Ratnakar Deole

**Affiliations:** a Oklahoma State University Center for Health Sciences, Tulsa, Oklahoma, USA; b Oklahoma State University, Stillwater, Oklahoma, USA; c University of West Alabama, Livingston, Alabama, USA; University of Maryland School of Medicine

## Abstract

Here, we report the draft genome sequence of Lactococcus lactis strain PrHT3, which was isolated from organic basil. This strain possesses one chromosome and two plasmids. This strain possesses potential probiotic characteristics.

## ANNOUNCEMENT

Lactic acid bacteria (LAB) are found in a variety of different environments, including plant materials and animals (1). Health benefits imparted by LAB with probiotic characteristics include antibacterial activity against pathogens, immune system stimulation, and improvement in inflammatory responses (2–4). Food and beverage market research reports estimate the probiotic market size by revenue to reach 69 billion dollars by 2023 (5). Here, we report the draft genome sequence of Lactococcus lactis (a member of the LAB) strain PrHT3, which was isolated from organic basil.

L. lactis strain PrHT3 was isolated from LAB isolated from grocery organic basil using de Man, Rogosa, and Sharpe (MRS) lactobacillus broth and agar for growth and was identified using 16S rRNA gene sequencing with an ABI 3730XL sequencer (6). Produce material was collected from a local grocery store in Tuscaloosa, Alabama, refrigerated at 4°C (i.e., storage), homogenized in MRS broth in sterile sampling bags, enriched at 30°C for 8 h, and plated on MRS agar plates using a sandwich overlay method described by Henning et al. (7). Bacterial colonies were separately streaked for establishment of pure cultures and then stored at −76°C in sterile MRS broth containing 10% glycerol. Bacterial culture material and identification information is available at https://doi.org/10.6084/m9.figshare.20497074.

For genomic DNA sequencing, L. lactis strain PrHT3 was grown overnight at 37°C in MRS broth, and genomic DNA was prepared using a Qiagen Genomic-tip 100/G kit. Paired-end libraries were prepared using the KAPA/Roche HyperPlus DNA library preparation kit (catalog number KK8512) and sequenced on an Illumina NextSeq 500 sequencer using the midoutput 150-cycle flow cell, which generated 288,757,304 total reads. The data generated were processed to obtain high-quality reads using bcl2fastq (Illumina Inc.), Trimmomatic v. 0.38 (8), FastQC v. 0.11.9 (9), and custom scripts (available at https://github.com/hoytpr/Lacto_genome). Subsequently, assembly of the genome and plasmids used 4 million randomly selected paired-end reads to create an average genomic read coverage of 216-fold. The initial *de novo* assembly using SPAdes v. 3.15.0 (10) produced long contigs for use with BLAST (11) to identify a reference genome (GenBank assembly accession number GCA_016028835.1). The SPAdes assembler was also used with the plasmid option to identify putative plasmid sequences. The assembly and plasmid sequences were visualized using Bandage v. 0.8.1 (12), and the presumptive plasmid nodes were extracted as fasta contigs. To separate plasmid reads from genome reads, the fasta contigs from Bandage were converted to a BLAST+ database (separately for each plasmid) and searched with all reads returning the read names. Read names for each plasmid were used to separate and remove plasmid reads from the genome reads. The subsequent genome-only reads were scaffolded to the reference genome and submitted as .bam files using Bowtie2 v. 2.4.2 (13). Default parameters were used for all software unless otherwise specified. The submission was annotated by PGAP v. 6.1 at NCBI (14).  Table 1 shows statistics for the sequencing data.

**TABLE 1:** Genomic features of Lactococcus lactis PrHT3

Genomic element	No. of reads	Size (bp)	No. of scaffolds	*N*_50_ (bp)	G+C content (%)	Mean coverage (×)
Chromosomal DNA	144,378,652	2,448,464	98	134,484	35.22	215
Plasmid 1 (plasmid 1ab)	571,732	12,772	8	4,564	31.69	3,357
Plasmid 2 (plasmid 2)	417,160	57,382	13	11,477	34.69	545

### Data availability.

The 16S rRNA gene sequence was deposited in the NCBI database under GenBank accession number OP090157. The draft whole-genome sequencing (WGS) project was deposited in the NBCI database under GenBank accession number JAHLJE000000000. The reads have been deposited in the SRA under accession number SRR14619658. The BioProject accession number is PRJNA731925.
